# Study of Association between Pre-Senile Cataracts and the Polymorphisms rs2228000 in XPC and rs1042522 in p53 in Spanish Population

**DOI:** 10.1371/journal.pone.0156317

**Published:** 2016-06-01

**Authors:** Gloria López Valverde, Elena Garcia Martin, José M. Larrosa Povés, Vicente Polo Llorens, Luis E. Pablo Júlvez

**Affiliations:** 1 Hospital Royo Villanova, Zaragoza, Spain; 2 Hospital Universitario Miguel Servet, Zaragoza, Spain; 3 Instituto de Investigación Sanitaria Aragón (IIS Aragon), Zaragoza, Spain; University of Hawaii Cancer Center, UNITED STATES

## Abstract

**Purpose:**

To determine if the presence of certain polymorphisms in the DNA repair gene XPC and the apoptosis inductor gene p53 is associated with pre-senile cataract development.

**Methods:**

We have performed a retrospective study over three groups of patients. The group with pre-senile cataract formed by 72 patients younger than 55 with cataract surgery. The group with senile cataract formed by 101 patients older than 55 with cataract surgery. The group without cataract was formed by 42 subjects older than 55 without lens opacities. We analyzed the presence of SNP rs2228000 from XPC and rs1042522 from p53; and the relationship between risk factors such as smoking, alcohol intake, hypertension or diabetes.

**Results:**

The comparison of the genotype distribution in XPC, within the different groups, did not show any statistically significant association in any of our analysis (p>0,05). The comparison of the genotype distribution in p53 within the different groups did not show any statistically significant association (p>0,05); except for the comparison between the pre-senile cataract group and the group with senile cataract where the genotype Pro/Pro (C/C) in the recessive inheritance model showed a higher risk for developing pre-senile cataract (p = 0,031; OR = 1.04–15.97). This association decreased when we performed the analysis adjusting by the studied risk factors (p = 0.056).

**Conclusions:**

Allelic variants in the gene XPC are not associated with an increased risk for developing pre-senile cataract. The presence of the genotype Pro/Pro in p53 might be associated with a major risk for developing pre-senile cataract.

## Introduction

Cataract consists in a loss of the lens transparency and it is considered to be the leading cause of treatable blindness worldwide [1]. It is usually caused by the physiological aging process [2]; however, a percentage of patients may develop cataracts in ages prior to senescence. Although its development is usually associated with aging, there are some conditions that may predispose to early cataract formation such as myotonic dystrophy, diabetes mellitus [3, 4], scleroderma, or metabolic diseases. Some drugs intake like corticosteroids can also predispose to the development of pre-senile cataracts [1, 5–7]. Nevertheless, there are certain cases of cataracts in young patients without any of these risk factors implicated.

Several theories have been proposed for cataract development, such as excess ultraviolet radiation (UV-r) [8, 9] or ionizing radiation [10, 11].

In our genome there are certain genes responsible for DNA repair to external aggressions. They encode the proteins that recognize genome cell damage and repair it. These proteins postpone cell replication until lesions have been repaired and otherwise they induce cell apoptosis. When these mechanisms fail, damage can be transmitted to progeny cells and begin processes such as carcinogenesis or cataract formation [12, 13]. XPC (Xeroderma pigmentosum complementation group C) have a role in DNA repair by nucleotide excision (NER), while p53 handles cell apoptosis when DNA repair mechanisms have failed.

XPC is a gene located in the short arm of the chromosome 3 (3p25). It encodes the protein XPC which function is to recognize DNA damage in the nucleotide excision repair (NER) [14, 15]. XPC also forms an initiation complex with HHRAD23 [16]. Its main function is to induce the recruitment and subsequent binding of other proteins to DNA. When the DNA damage is recognized, it is produced a conformational change in the XPC protein so that the aromatic residues of the amino acids join the nucleotides unpaired in front of the damage. This is essential for the formation of open protein complexes and their repair [17]. XPC contains several binding domains: for DNA binding and binding to hHR23B, Centrin2, 8-oxoguanine glycosylase (OGG1), or p62/SQSTM1 and transcription factor II H (TFIIH). TFIIH is a multifunctional transcription initiation factor but is also a core NER component [18]. In addition to its role in DNA repair, XPC also play an important role in cell-cycle arrest and activation of the p53 pathway [19]. Moreover XPC interacts with negative regulation factor in p53, MDM2. When UV damage is produced the association between MDM2 and XPC in vivo is attenuated, likely due to reduced MDM2 levels [20]. Alterations in the XPC gene prevent an adequate synthesis of the protein, whereby the DNA nucleotide excision repair pathway is altered. The damaged DNA can accumulate and carcinogenesis may occur [17].

p53 is located in the short arm of chromosome 17 (17p13) and it encodes a nuclear transcription factor. It encodes the protein p53, which acts on genes that regulate cell replication cycle. In situations of DNA damage, p53 stops the cell cycle until the DNA is repaired or until cell death is provoked. In that way it prevents mutated cells proliferation [21]. In unstressed cells, p53’s activity is controlled by its negative regulator MDM2.The protein p53 has five functional domains responsible for regulating cellular response to DNA damage [22, 23]. It has a specific acidic transactivation domain at the N-terminus that interacts with transcriptional co-activators, co-repressors and general transcription factors. Among other proteins it interacts with p62, subunit of the transcriptional factor TFIIH which role is primordial to DNA repair via nucleotide excision (NER). XPC has a specific acidic fragment that also binds to p62 [24]. P53 also has a region rich in proline residues important for p53-mediated apoptotic processes [25]. The DNA binding domain (DBD) is located in the central region of the protein. The first step in p53-mediated transcription is the binding of the protein to its recognition site in DNA [26]. The protein p53 is also involved in the DNA damage repair by activating the transcription of several genes as ribonucleotide reductase p53R2, XPC or GADD45, thus inhibiting DNA synthesis and stimulating the nucleotide excision repair [27, 28].

There are evidences that both proteins (P53 and XPC) are implicated with NER. In fact the global genomic repair (GGR) is a p53-mediated process and p53 regulated the basal and inducible levels of XPC and p48, both proteins are essential for GGR [29].

The aim of this study is to assess whether gene polymorphisms rs2228000 in XPC and rs1042522 in p53, may be involved in the development of cataracts in early ages in a group of population from Salamanca. This is the first study conducted to connect these genes polymorphisms with pre-senile cataract formation.

## Methods

Our research has been approved by the Ethics Committee of the Clinic Hospital of Salamanca and adhered to the principles of the Declaration of Helsinki.

Written informed consent was obtained from all our patients.

Based on a preliminary study performed by our group, we calculated the size sample needed to detect differences between groups, with an α risk of 5% and β risk of 10% (i.e., with a power of 90%). There were 38 subjects in each group. In order to obtain a sufficient sample of patients, to allow us a thorough study of the natural history of disease, 72 subjects were included in the group with pre-senile cataract, 101 patients in the group of senile cataracts and 42 in the group of subjects without cataract.

To form the group with pre-senile cataract, we selected, sequentially, a sample of 72 patients who had undergone cataract surgery at the Clinic Hospital of Salamanca from January 2007 to December 2012. This group included all adult patients younger than 55 years old who had undergone cataract surgery. We excluded those patients who had cataracts secondary to trauma, uveitis, previous eye surgery, steroids intake and patients with systemic diseases associated wit cataracts such as Steinert's syndrome, galactosemia, Lowe syndrome, homocystinuria, Alport syndrome, Marfan syndrome, Weil Marchesani syndrome, Peters anomaly, connective tissue diseases such as Ehlers-Danlos syndrome, Down syndrome and Cockayne syndrome.

To form the senile cataract, we selected 101 patients older than 55 years old who underwent cataract surgery. We also excluded those patients whose cataracts are secondary to the above mentioned diseases; and to form the group with no cataracts, we included 42 patients older than 55 who had not yet developed cataracts. In this group we excluded all those patients who presented any type of valuable lens opacity by LOCS III system [30], or those with refractive changes resulting from lens aging.

We analyzed variables such as the presence of risk factors (RF) which may be associated with cataracts development like the presence of diabetes mellitus, arterial hypertension, smoking at least 10 cigarettes a day for 5 or more years, alcohol intake of at least one unit alcohol at least two times a week. We also analyzed the genotypic distribution of the polymorphisms rs2228000 in XPC and rs1042522 in p53.

We performed a complete anamnesis to all the subjects. This anamnesis included past medical history, age and gender, and potential risk factors such as the presence of diabetes, arterial hypertension, smoking or alcohol intake. We also performed a complete ophthalmic examination, consisting on refractive error calculation, visual acuity measurement (VA) with Snellen chart, intraocular pressure measurement (IOP) by Goldman applanation tonometry, slit lamp examination to assess the degree of lens opacity using the LOCS III system [30], biometrics and funduscopy. The criteria to be included for cataract surgery was to have a VA under 20/40 without any ophthalmic diseases which justified a VA loss.

Informed consent was obtained from all the participants included in the study. After that, we took a sample of peripheral blood, in order to isolate the cellular DNA following the standard protocols. The allelic discrimination of the single nucleotide polymorphism (SNP) rs2228000 in XPC was performed by PCR using Taqman probes; ID test C_16018061_10. The study was performed in a thermocycler (Step One Plus Real-Time PCR system, Applied Biosystems, Foster City CA) where we introduced a 96 cell-container. We mixed one microliter of DNA sample from each patient; 5 ml of the commercial compound PCR (Taqman ™), which provides the necessary enzyme for amplification (Taq polymerase); 0.25 ml of the commercial compound which contains the first primer oligonucleotide "forward", the first primer oligonucleotide "reverse" and the probes labeled with VIC and FAM dyes; and 4.25 ml of distilled water. After an initial denaturation of the mixture at 95°C for three minutes followed by 35 cycles of denaturation at 94°C for 10 seconds, an annealing procedure at 58°C for 20 seconds and an elongation at 72°C for 40 seconds the amplification was done. In all PCR reactions a final elongation of 7 minutes at 72°C was performed.

**Fig 1:**
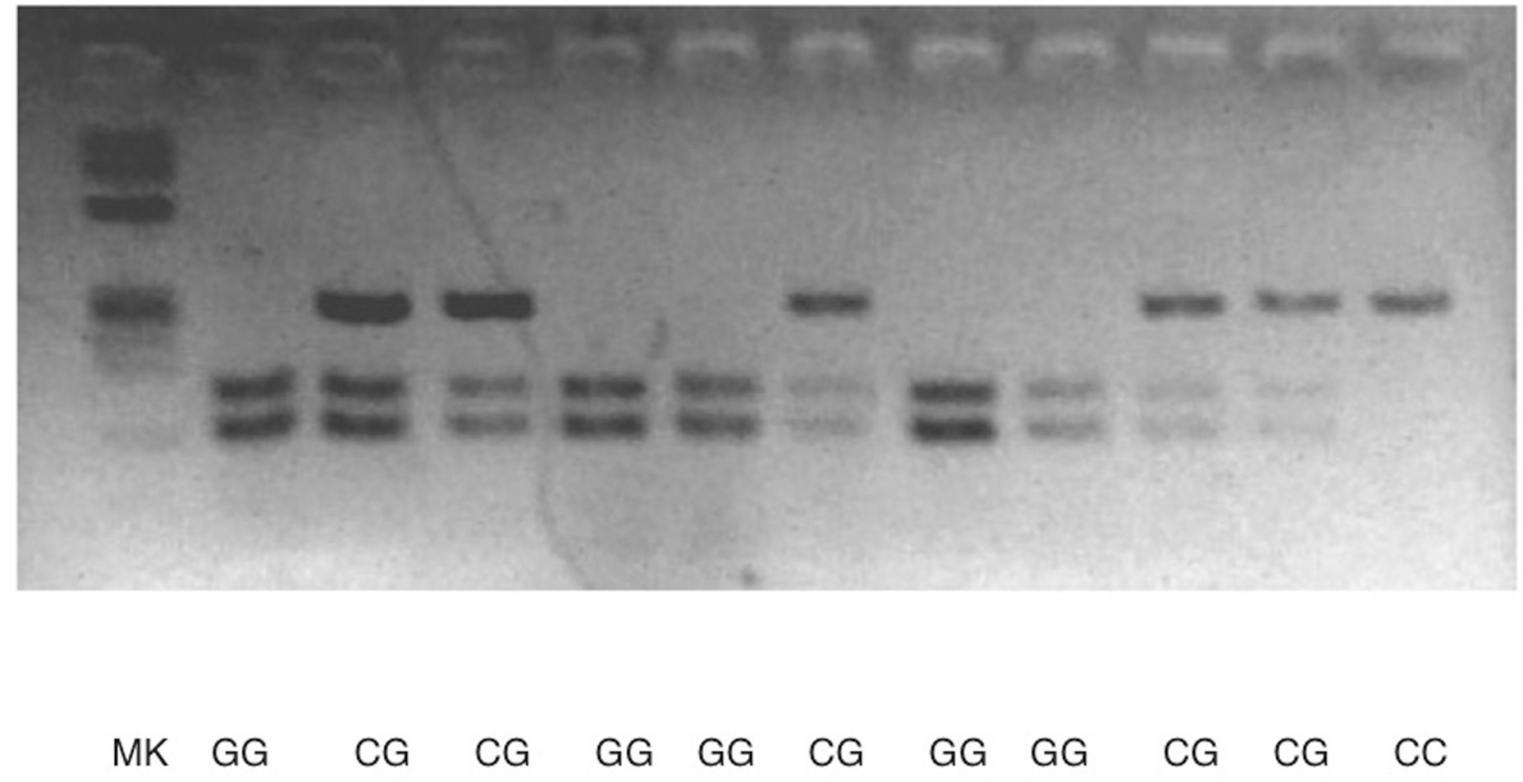
Agarose picture from rs1042522 p53 analysis.

We used restriction endonucleases to analyze p53. These enzymes recognize specific DNA sequences and cleave it at this point. The study of the restriction fragments length polymorphism (RFLP) is a technique used to discriminate different genes alleles, analyzing the size of the fragments generated after the DNA digestion with restriction enzymes. Digestions were performed by incubating 12μL of the PCR product plus 1 mL of the selected restriction endonuclease, BstUI (Bsh12361, Fermentas), and 7 mL of water, used as the digestion buffer-specific endonuclease or as universal buffer. It is placed at the specific temperature for 5 to 7 hours. The fragments obtained after the digestion were separated by electrophoresis in an agarose gel at 1% or 3% stained with Syber-safe ®. In all gels we included the size marker. In order to monitor the DNA migration at the agarose gel two dyes Xylene cyanol and bromophenol blue were included in the buffer. We accomplish a digital photograph under ultraviolet light using the Kodak Science ID program (Kodak, SA). (Fig 1)

This is a one-center, retrospective, case-control study approved by the Ethics Committee of the Clinic Hospital of Salamanca and adhered to the principles of the Declaration of Helsinki.

The distribution of the percentages was calculated for each qualitative variable. For the variable age we calculated indicators of central tendency (mean or median) and dispersion (Standard deviation or percentiles). Differences in risk factors frequencies between the different groups (Case-Control) were investigated using established hypothesis comparing proportions. Genetic association analysis was performed after verifying whether polymorphisms fulfilled Hardy-Weinberg equilibrium. The polymorphisms association magnitude with the disease was quantified by calculating odds ratios (OR) of each genotype with regard to the reference group (control group).

In a previous study, the comparison of environmental and acquired risk factors (smoking, alcohol, diabetes, hypertension) between the different groups showed that smoking and alcohol intake are related to the presence of pre-senile cataracts with a significant association (p<0.01). Likewise, alcohol intake also seems to influence the formation of senile cataract type (p = 0.027). Hypertension was more frequent in the groups formed with patients older than 55 years old. Hence we performed a regression model adjusting for these environmental and acquired risk factors, logistic regression models adjusting for risk factors with statistical significance in the two-dimensional analysis, and depending on the model of inheritance: dominant, recessive and codominant alleles. The accuracy of the association was expressed through a 95% of confidence interval (CI). The results were considered statistically significant when the p-value was less than or equal to 0.05. The statistical analysis was performed by an independent person using the Free Software package SNPAsocc R.

## Results

For this study we have established three groups of subjects: the group with pre-senile cataract (adults younger than 55 years old diagnosed and treated of cataract); the group with senile cataract (adults older than 55 who had undergone cataract surgery), and the group with no cataract (adults older than 55 who had no lens opacification). The average age of the group with pre-senile cataracts was 49.01± 7.26 years (range 26–55 years); in the group with senile cataract was 73.79 ± 6.53 years (range 60–91 years) and in the group with no cataracts was 67.10 ± 4.64 years (range 57–77 years).

The SNP rs2228000; c.1385 G>A in the gene XPC produces a change of an adenine (A) for a guanine (G). The comparison of the distribution of genotypes and alleles of this SNP between the group with pre-senile cataract and the group with senile cataract showed no significant differences (p>0.05). We also performed the analysis using the regression model adjusted to the studied risk factors (smoking and alcohol); and we obtained no statistically significant differences in any model of inheritance (p> 0.05) (Table 1).

**Table 1:** Polymorphism genotype distribution (rs 2228000) in XPC gene in pre-senile and senile cataracts groups.

Models of genetic heritage	Pre-senile cataracts group	Senile cataracts group	OR	p
Codominant				
G/G	35(48,6%)	57(56,4%)	1,00	0,260
G/A	30(41,7%)	40(39,6%)	1,22(0,65–2,30)	0,372*
A/A	7 (9,7%)	4 (4,0%)	1,31(0,61–2,83)*	
			2,85 (0,78–10,44)	
			2,96(0,58–15,04)*	
Dominant				
G/G	35(48,6%)	57(56,4%)	1,00	0,309
G/A-A/A	37(51,4%)	44(43,6%)	1,37 (0,75–2,51)	0,310*
			1,46 (0,70–3,04)*	
Recessive				
G/G-G/A	65(90,3%)	97(96,0%)	1,00	0,129
A/A	7(9,7%)	4 (4,0%)	2,61 (0,73–9,28)	0,223*
			2,64(0,54–13,00)*	

*: Values according to regression model adjustment of risk factors

The comparison of the genotype distribution for the SNP rs2228000 in XPC between the group with pre-senile cataract and the group with no cataracts showed no statistically significant differences (p>0.05). When performing the alleles’ grouping analysis no differences were observed. We also performed the analysis using the regression model adjusted to risk factors; and found no statistically significant differences (p>0.05) (Table 2).

**Table 2:** Polymorphism genotype distribution (rs 2228000) in XPC gene in pre-senile cataracts group and the group with no cataract.

Models of genetic heritage	Pre-senile cataracts group	Group with no cataract	OR	p
Codominant				
G/G	35(48,6%)	25(59,5%)	1,00	0,214
G/A	30(41,7%)	16(38,1%)	1,34(0,60–2,96)	0,368*
A/A	7(9,7%)	1 (2,4%)	1,54(0,55–4,36)*	
			5,00 (0,58–43,24)	
			4,70(0,37–59,68)*	
Dominant				
G/G	35(48,6%)	25(59,5%)	1,00	0,259
G/A-A/A	37(51,4%)	17(40,5%)	1,55 (0,72–3,36)	0,273*
			1,75(0,64–4,77)*	
Recessive				
G/G-G/A	65(90,3%)	41(97,6%)	1,00	0,110
A/A	7(9,7%)	1(2,4%)	4,42 (0,52–37,21)	0,250*
			3,90(0,32–47,14)*	

*: Values according to regression model adjustment of risk factors

The comparison of the genotype distribution for the SNP rs2228000 in XPC between the group with senile cataract and the group with no cataract showed no statistically significant differences; neither in the alleles’ grouping analysis. When performing the analysis adjusted to environmental and acquired risk factors no differences were observed (p>0.05) (Table 3).

**Table 3:** Polymorphism genotype distribution (rs 2228000) in XPC gene in the senile cataracts group and the group with no cataract.

Models of genetic heritage	Senile cataracts group	Group with no cataract	OR	p
Codominant				
G/G	57(56,4%)	25(59,5%)	1,00	0,863
G/A	40(39,6%)	16(38,1%)	1,10(0,52–2,31)	0,923*
A/A	4 (4,0%)	1 (2,4%)	1,16(0,55–2,48)*	
			1,75 (0,19–16,50)	
			0,98(0,08–11,33)*	
Dominant				
G/G	57(56,4%)	25(59,5%)	1,00	0,733
G/A-A/A	44(43,6%)	17(40,5%)	1,14 (0,55–2,36)	0,706*
			1,15(0,55–2,42)*	
Recessive				
G/G-G/A	97(96,0%)	41(97,6%)	1,00	0,627
A/A	4 (4,0%)	1(2,4%)	1,69 (0,18–15,59)	0,947*
			0,92(0,08–10,45)*	

*: Values according to regression model adjustment of risk factors

The SNP rs1042522 (c.215 C>G) in the gene p53 produces a change of a citosine (C) by a guanine (G) what produces the change of a proline (Pro) by an arginine (Arg) in the 72th codon of the protein. The comparison of the genotypes and alleles distribution of this polymorphism between the group with pre-senile cataract and the group with senile cataract showed a higher frequency of the genotype CC in the group with pre-senile cataract in the recessive inheritance model (p = 0,03082; OR = 1,02–15,97). However, when we performed the regression analysis adjusted to the studied risk factors the statistical signification decreased (p = 0,056) (Table 4).

**Table 4:** Polymorphism genotype distribution (rs 1042522) in P53 gene in pre-senile and senile cataracts groups.

Models of genetic heritage	Pre-senile cataracts group	Senile cataracts group	OR	p
Codominant				
G/G	41(56,9%)	57(56,4%)	1,00	0,073
G/C	23(31,9%)	41(40,6%)	0,78(0,41–1,49)	0,141*
C/C	8(11,1%)	3(3,0%)	0,81(0,37–1,77)*	
			3,71 (0,93–14,83)	
			4,55(0,80–25,86)*	
Dominant				
G/G	41(56,9%)	57(56,4%)	1,00	0,947
G/C-C/C	31(43,1%)	44(43,6%)	0,98 (0,53–1,80)	0,959*
			1,02(0,49–2,13)*	
Recessive				
G/G-G/C	64(88,9%)	98(97,0%)	1,00	0,031
C/C	8(11,1%)	3 (3,0%)	4,08 (1,04–15,97)	0,056*
			4,93(0,89–27,37)*	

*: Values according to regression model adjustment of risk factors

The comparison of the genotypes and alleles distribution of the SNP rs1042522 in p53 between the pre-senile cataract group and the group with no cataract showed no statistical significant association, neither when we performed the analysis adjusted to the studied risk factors (p>0,05) (Table 5).

**Table 5:** Polymorphism genotype distribution (rs 1042522) in P53 gene in the pre-senile cataract group and the group with no cataract.

Models of genetic heritage	Pre-senile cataracts group	Group with no cataracts	OR	p
Codominant				
G/G	41(56,9%)	20(47,6%)	1,00	0,506
G/C	23(31,9%)	18(42,9%)	0,62(0,28–1,41)	0,519*
C/C	8(11,1%)	4(9,5%)	0,55(0,19–1,57)*	
			0,98 (0,26–3,63)	
			0,66(0,11–4,11)*	
Dominant				
G/G	41(56,9%)	20(47,6%)	1,00	0,335
G/C-C/C	31(43,1%)	22(52,4%)	0,69 (0,32–1,48)	0,259*
			0,57(0,21–1,52)*	
Recessive				
G/G-G/C	64(88,9%)	38(90,5%)	1,00	0,788
C/C	8(11,1%)	4 (9,5%)	1,19 (0,33–4,21)	0,842*
			0,83(0,14–4,97)*	
			0,83(0,14–4,97)*	

*: Values according to regression model adjustment of risk factors

The comparison of this SNP genotypes distribution between the group with senile cataract and the group with no cataract showed no statistical significant association in any of the analysis performed (p>0,05) (Table 6).

**Table 6:** Polymorphism genotype distribution (rs 1042522) in P53 gene, in the group with senile cataract and the group with no cataract.

Models of genetic heritage	Senile cataracts group	Group with no cataracts	OR	p
Codominant				
G/G	57(56,4%)	20(47,6%)	1,00	0,246
G/C	41(40,6%)	18(42,9%)	0,80 (0,38–1,70)	0,307*
C/C	8(3,0%)	4(9,5%)	0,81(0,38–1,73)*	
			0,26 (0,05–1,28)	
			0,29(0,06–1,43)*	
Dominant				
G/G	57(56,4%)	20(47,6%)	1,00	0,335
G/C-C/C	44(43,6%)	22(52,4%)	0,70 (0,34–1,44)	0,365*
			0,71(0,34–1,48)*	
Recessive				
G/G-G/C	98(97,0%)	38(90,5%)	1,00	0,116
C/C	3(3,0%)	4 (9,5%)	0,29 (0,06–1,36)	0,151*
			0,32(0,07–1,52)*	

*: Values according to regression model adjustment of risk factors

## Discussion

The gene XPC is located in the short arm of chromosome 3 (3p25). It encodes a protein involved in the DNA nucleotide excision repair (NER) by recognizing DNA damage [21, 31]. This protein intervenes with other proteins, XPA and a complex formed by three proteins known as replication protein A (RPA), during DNA damage recognition [32]. XPC also activates the p53 pathway and induces cell-cycle arrest [19]. Some studies have described the relationship between the complex p53-hHR23B and XPC in the modulation of initial steps of NER [33].

We study the SNP rs2228000 (c.1385 G> A), which produces a change of an adenine (A) by a guanine (G). SNP rs2228000 is located in the interaction domain of XPC with RAD23B, although its functional significance remains unclear. It is possible that this variant, may alter NER capacity, thereby modulating cancer and aging susceptibility. Nevertheless we did not find any statistically significant differences in this polymorphism genotypes distribution in any of our analysis. Since there are no studies linking XPC with cataract formation, our results need to be confirmed.

Cellular senescence is an antiproliferative program that leads to permanent growth arrest. Senescent cells are irreversibly arrested in G1/G0 phase of the cell cycle and lose the ability to respond to growth factors. They maintain metabolic activity for long periods of time and become resistant to apoptosis [34]. This growth arrest is mediated by interplay between multiple pathways, most notably, the p16/Rb and p53/21 signal pathways. Currently, most of the cellular senescence regulation molecules identified have been shown to play roles in p16- or p53-dependent manners [35]. As we have previously described, p53 has five functional domains.

Most mutations in p53 occur in the central DNA-binding domain (DBD) due to p53’s major function as transcription factor that controls the expression of a multiple genes that regulate apoptosis, senescence, cell-cycle arrest and DNA repair. Functional mutations that alter the transcriptional capacity of the p53 master gene have been identified as super-transactivating, change-in-spectrum or overall downward modulation of transactivation. For example, change-in-spectrum mutants may be capable of regulating genes containing a strong response element, such as p21, but unable to regulate those with a weak response element, such as Bax. This is consistent with the observation of mutant p53s that still induce cell cycle arrest, yet lose the ability to activate apoptosis [36, 37]. Some mutants of p53 are temperature sensitive, such as Arg175, Gly245, Arg248, Arg249, Arg273, and Arg282, which have been widely described in association with human cancer. Nevertheless we have not found any reports of temperature-sensitive mutants in p53 related with senescence or aging [38, 39].

Because of the relationship of p53 with senescence and its association with the gene XPC we decided to add the polymorphism in the 72th codon in p53 gene to our study. Besides some authors have studied the association between oxidative damage and an increase of apoptosis in the lens cells [40, 41]. This SNP rs1042522 (c.215 C> G) consists of change of a cytosine (C) by a guanine (G) what produces a change of Proline (Pro) by an Arginine (Arg) at the codon 72 of the protein. This polymorphism P.Arg72Pro is located in the region rich with proline residues, which is involved in the induction of apoptosis [42]. The arginine allele has a great ability to induce apoptosis due to the activation of transcriptional apoptotic genes. It also can induce apoptosis by mechanisms independent of its transcriptional activity. On the other hand, the proline allele has less apoptotic capacity so it induces the cell cycle arrest in G1 phase in response to DNA damage [43–45].

Several studies have related this polymorphism with ophthalmological diseases such as retinal vitreous proliferation following retinal detachment [46] or with glaucoma [47, 48]. Ji et al determined that p53 regulates αA- and βA3 / A1–crystallin genes to modulate cell differentiation genes in lens [49]. We have not found any articles about the association of this polymorphism and cataracts development. Qin et al showed that changes in the micro—125b cro ARN inhibit apoptosis of lens epithelial cells via p53 leading to the development of senile cataracts [50].

The comparison of the genotypes and alleles distribution for the SNP between the pre-senile cataract group and the senile cataract group showed significant association in the recessive model, where a higher percentage of patients with pre-senile cataract have homozygous genotype Pro/Pro. It represents an increased risk for developing pre-senile cataract of 4,083 (p = 0,03082; OR = 1,02–15,97). However, when we performed the analysis adjusted to the risk factors, the statistical significance decreased (p = 0.05614; OR = 0.89–27.87) (Table 4). The decrease in the apoptotic activity at lens cells could lead to an accumulation of cell damage and be the cause of cataract development at a younger age, justifying the results obtained in our series. This is the first study that shows that the individuals carrying the Pro allele, which is associated with lower apoptosis activity, are more likely to develop cataracts at an early age. This may be because damaged lens cells, either by ultraviolet radiation or by oxidative mechanisms, are more resistant to apoptosis and remain in the lens damaged inducing cataract formation.

In summary, SNP rs2228000 in XPC is not associated with pre-senile cataract development in our study. The presence of the genotype Pro/Pro in rs1042522 of p53 might be associated with a major risk for developing pre-senile cataract. Further investigations over these two genes would be useful to reject or accept their association with pre-senile cataract development.
