# Correction: Study of Association between Pre-Senile Cataracts and the Polymorphisms rs2228000 in XPC and rs1042522 in p53 in Spanish Population

**DOI:** 10.1371/journal.pone.0171395

**Published:** 2017-01-26

**Authors:** Gloria López Valverde, Elena Garcia Martin, José M. Larrosa Povés, Vicente Polo Llorens, Javier Fernández Mateos, Luis E. Pablo Júlvez, Rogelio González Sarmiento

Dr. Javier Fernández Mateos is not included in the author byline. He should be listed as the fifth author and affiliated with the Molecular Medicine Unit, Department of Medicine, Institute of Molecular and Cellular Biology of Cancer and Institute of Biomedical Research of Salamanca, University of Salamanca-University Hospital of Salamanca CSIC, Salamanca, Spain. The contributions of this author are as follows: performed the experiments.

Dr. Rogelio González Sarmiento is not included in the author byline. He should be listed as the seventh author and affiliated with the Molecular Medicine Unit, Department of Medicine, Institute of Molecular and Cellular Biology of Cancer and Institute of Biomedical Research of Salamanca, University of Salamanca-University Hospital of Salamanca CSIC, Salamanca, Spain. The contributions of this author are as follows: conceived and designed the experiments, and provided resources.

The correct citation is: López Valverde G, Garcia Martin E, Larrosa Povés JM, Polo Llorens V, Fernández Mateos J, Pablo Júlvez LE, et al. (2016) Study of Association between Pre-Senile Cataracts and the Polymorphisms rs2228000 in XPC and rs1042522 in p53 in Spanish Population. PLoS ONE 11(6): e0156317. doi:10.1371/journal.pone.0156317
